# Effectiveness of 8-Week Exercise Programs in Improving Menstrual Characteristics in Female Adolescents in Saudi Arabia

**DOI:** 10.3390/healthcare12192005

**Published:** 2024-10-07

**Authors:** Asma Alonazi, Norah Alqashami, Rand Alkhamis, Aseel Almutairi, Athil Arishi

**Affiliations:** 1 Department of Physical Therapy and Health Rehabilitation, College of Applied Medical Sciences, Majmaah University, Al-Majmaah 11952, Saudi Arabia; 2 Department of Physical Therapy and Health Rehabilitation, Prince Nasser bin Saad Al-Sudairi Hospital, Al Ghat 15912, Saudi Arabia; 3 Department of Physical Therapy and Health Rehabilitation, College of Applied Medical Sciences, King Abdullah bin Abdulaziz University Hospital, Riyadh 13412, Saudi Arabia; 4 Program of Physical Therapy, Department of Medical Rehabilitation Sciences, College of Applied Medical Sciences, King Khalid University, Abha 62521, Saudi Arabia

**Keywords:** adolescents, exercise, menstrual cycle, pain, attitude

## Abstract

**Background:** This study aimed to evaluate the effectiveness of 8-week exercise programs (aerobic, stretching, and aerobic plus stretching) in improving menstrual characteristics in female adolescents in Saudi Arabia. **Methods:** In this quasi-experimental study, seventy-eight healthy female adolescents aged 12–18 from secondary and high schools in Riyadh participated. Participants were divided into three groups and subjected to different types of exercises (aerobic, stretching, and aerobic plus stretching) for 20–30 min, three times a week. Menstrual pain, stress, and attitudes were assessed using the Numerical Rating Scale, Adolescent Stress Questionnaire Short Version, and Adolescent Menstrual Attitude Questionnaire, respectively. Assessments were conducted before the intervention and after the third menstrual period post-intervention. **Results:** The aerobic exercise group experienced a significant reduction in the mean length of the menstrual cycle (*p* = 0.025). The aerobic plus stretching group had the most significant reduction in mean menstrual pain scores (*p* < 0.0001). Stress scores were significantly reduced in the stretching group (*p* < 0.0001). **Conclusion:** We conclude that combining aerobic and stretching exercises significantly reduces menstrual pain, while aerobic exercise decreases menstrual cycle length and pain.

## 1. Introduction

Adolescence is a phase in life characterized by the transition from childhood to adulthood. During this period, individuals experience physical, psycho-cognitive, and social changes. For young female adolescents, menarche (the first occurrence of menstruation) is the most prominent change [1]. Menstruation is a cyclical biological phenomenon in women of reproductive age, associated with various physical, emotional, cognitive, and behavioural symptoms [2]. Approximately 50–90% of women experience severe painful menstruation [3]. About 10–15% of them suffer from severe symptoms of dysmenorrhea, resulting in a functional loss of days each month [4].

Culture substantially influences menstrual stigma, determining perceptions and attitudes towards menstruation [5]. Adolescent girls, often unaware of proper menstrual hygiene, frequently encounter negative cultural attitudes and engage in unsanitary menstruation practices at home, school, or in other public settings. This is due to a combination of distorted information, fear of societal judgment, insufficient facilities, and a lack of absorbent supplies [6]. In Saudi Arabia, patients often do not seek medical help in menstruation-related conditions such as dysmenorrhea [7]. Although the role of culture in this has not been studied in Saudi Arabia, either quantitatively or qualitatively, one possible explanation could be the societal expectations of modesty and privacy, along with the restricted public discussion on menstruation.

Several factors influence the length and regularity of the menstrual cycle, including sociodemographic characteristics, psychological and psychosocial well-being, sleep pattern, physical activity, and dietary habits [8]. Numerous studies have reported high levels of stress during painful menstruation and identified stress as a significant contributor to irregular menstruation [9,10,11]. Physiologically, stress heightens sympathetic nervous system activity, which exacerbates uterine muscle contractions and increases menstruation-related symptoms [12,13].

Research suggests that physical exercises such as strength training, yoga, aerobic activity, and the Kegel manoeuvre significantly reduce the intensity of menstrual pain and related symptoms [14,15]. Over the past two-to-three decades, a physically active lifestyle and regular exercise have been regarded as ideal methods for the prevention and treatment of menstrual pain and symptoms such as dysmenorrhea [14,16,17,18]. Regular exercise significantly affects the release of neurotransmitters like endorphins, dopamine, oestrogen, and endogenous opiate peptides, while suppressing prostaglandins, all of which work together to enhance the pain threshold [19]. Additionally, regular exercise elevates mood, improves behaviour, and reduces negative thoughts [20].

Several research studies have reported the positive influence of exercise on menstruation-related issues, such as primary dysmenorrhea [14,15]. A study by Tsai et al. (2024) confirmed the effectiveness of both stretching and aerobic exercises in decreasing the severe symptoms of dysmenorrhea [15]. Another study from India by Vaghela et al. (2019) found that both aerobic workouts and yoga movements were effective in alleviating premenstrual syndrome; however, yoga was identified as more effective in reducing the symptoms compared to aerobic workouts [14].

Given the encouraging effects of exercise on health and well-being, the impact of different types of exercise on menstruation-related issues should be further explored. Investigating the role of various exercises and their effects on menstruation is important for optimizing women’s health and managing menstrual symptoms, especially in Saudi Arabia, where the topic remains underdiscussed. To the best of our knowledge, no study has yet assessed and compared the impact of aerobic, stretching, and aerobic plus stretching exercises on menstrual cycle dynamics, including menstrual pain, psycho-cognitive health, and attitude towards menstruation in female adolescents. The authors hypothesized that different types of exercise would be effective in reducing and managing the menstrual symptoms. Therefore, this study aimed to compare the effectiveness of 8-week exercise programs (aerobic, stretching, and aerobic plus stretching) on menstrual cycle characteristics in female adolescents in Saudi Arabia.

## 2. Materials and Methods

### 2.1. Ethics Approval

The study proposal was reviewed by the Institutional Review Board (IRB) of the Deanship of Scientific Research, Majmaah University, Research Ethics Committee, Al Majma’ah, Saudi Arabia, Reference # MUREC-Feb.12/COM-2022/7-4. Before enrolment, informed consent was obtained from the parents, and assent was taken from the participants. The anonymity and confidentiality of the participants was protected throughout the study.

### 2.2. Study Design, Setting, and Participants

The study employed a quasi-experimental design involving 78 healthy female adolescents aged from 12 to 18 years, recruited from two public secondary and high schools in Riyadh city, Saudi Arabia. Participants were evenly divided into three groups and subjected to 8-week exercise programs—either aerobic, stretching, or aerobic plus stretching—comprising three sessions per week, each lasting 20–30 min.

Inclusion criteria included healthy female adolescents within the specified age range (12–18 years), free from painkiller medication, and without musculoskeletal or neurological disorders. Exclusion criteria included a history of compulsory pain-relieving drug use, symptoms of vaginal yeast infection (such as tingling, itching, discharge), and adolescents with a body mass index (BMI) ≥ 95th percentile.

To minimize bias, investigators were blinded to group allocation throughout both the experiment and analysis phases. However, randomization was not utilized for assigning participants to groups; instead, allocation was sequential, ensuring transparency and consistency in group assignment across the study.

### 2.3. Study Procedure and Data Collection

The study started with the process of obtaining informed consent. Consent forms were initially distributed to the school principal for parental approval. During the initial visit, signed consent forms were collected, and participants were interviewed in person to obtain their assent. Following this, demographic information—including age, height (cm), weight (kg), body mass index (BMI; kg/m^2^), menstrual cycle length (days), menstrual bleeding heaviness, and physical activity level—was recorded.

Subsequently, participants completed pre-intervention questionnaires, including the Numerical Rating Scale (NRS), Adolescent Stress Questionnaire Short Version (ASQ-S), and Adolescent Menstrual Attitude Questionnaire (AMAQ). The NRS is a widely used tool for assessing pain intensity, with higher scores indicating greater pain. The ASQ-S measures stress level experienced, with higher scores reflecting greater stress levels, while the AMAQ assess attitudes towards menstruation, with higher scores representing more negative or problematic attitudes, and lower scores indicating more positive or accepting attitudes.

Each participant was then assigned to one of three exercise groups: aerobic exercise, stretching, or a combination of both. The exercise program, supervised by physical therapists, spanned eight weeks with sessions conducted three times a week, each lasting approximately 30 min. The exercises were carefully designed to be age-appropriate, ensuring safety and effectiveness for the adolescent participants. Throughout the program, participants were closely monitored to ensure adherence and to address any issues that arose during the sessions.

Following the eight-week exercise program, the same questionnaires (NRS, ASQ-S, and AMAQ) were re-administered to evaluate the intervention’s effectiveness by comparing the pre- and post-intervention data.

### 2.4. Eight-Week Exercise Program

#### 2.4.1. Aerobic Exercises

The aerobic exercise was conducted three times per week for 30 min over eight weeks. Each session included warm-up movements (head movements, traction, shoulder rotation, and balance), kinetic movements (arm rotation and elongation and upper rotation), and cool-down movements (resting and seated movements to return to the initial state) [21].

#### 2.4.2. Stretching Exercises

The upper and lower body stretching exercise was also conducted three times per week for 30 min over eight weeks. The exercises included biceps stretching, shoulder stretching, triceps stretching, sideways neck stretching, upper back stretching, chest stretching, wrist stretching, calf stretching, hamstring and lower back stretching, abdominal stretching, glutes and abductors stretching, groin and adductors stretching, quadriceps stretching, and full-body stretching [21].

#### 2.4.3. Both Aerobic and Stretching Exercises

These combined exercises included both the aerobic exercises and the upper and lower body stretching exercises detailed above. The combined regimen was performed three times per week for 30 min over eight weeks [21].

### 2.5. Outcome Measures

#### 2.5.1. Numerical Rating Scale (NRS)

The NRS is a tool that uses numbers to rate pain intensity. It employs a defined scale and asks individuals to verbally assign a number that match their pain intensity, place a mark on the number, or point to the number. The scale ranges from 0 to 10, where zero indicates the absence of pain and 10 represents the worst possible pain [22].

#### 2.5.2. Adolescent Stress Questionnaire Short Version (ASQ-S)

The ASQ-S is a 27-item self-report questionnaire that measures stress across nine of the original ten ASQ domains: home life, school attendance, performance, romantic relationships, peer pressure, school–leisure conflict, teacher interaction, future uncertainty, and finance. For each domain, participants are asked, ‘How stressful do you find it?’ (e.g., teacher interaction). The level of stress is measured on a 5-point Likert scale where 1 reflects ‘not stressful at all’ and 5 reflects ‘very stressful’ [23].

#### 2.5.3. Adolescent Menstrual Attitude Questionnaire (AMAQ)

The AMAQ is a 58-item measure that assesses adolescents’ attitude toward menstruation post-menarche. It evaluates five domains: a negative response to menstruation, a natural/accepting response to menstruation, an enthusiastic response to menstruation, a response to menstrual symptoms, and managing menstruation. This questionnaire was culturally adapted to fit the preferences of the Saudi culture [24].

### 2.6. Statistical Analyses

Statistical Package for Social Sciences (SPSS) for Windows, version 24.0 (IBM Corp., Armonk, NY, USA) was used for data analyses. Data normality was assessed using the Shapiro–Wilk test. Depending on the normality of the data, quantitative continuous variables were expressed as means ± standard deviations (SDs) or median and interquartile range (IQR), while qualitative categorical variables were expressed as numbers and percentages. Analysis of variance (ANOVA) was conducted to compare mean differences among the exercise groups. To determine between-group differences for post-intervention scores, analysis of covariance (ANCOVA) was performed, using pre-intervention scores and BMI used as the covariate. A *p* value of < 0.05 was considered statistically significant. A post hoc power analysis was conducted using Stata Version 17.0. The analysis focused on outcomes related to pain, stress, and attitude across the three exercise groups (aerobic, stretching, and aerobic plus stretching).

## 3. Results

### 3.1. Characteristics of Study Participants

Seventy-eight secondary and high school female adolescents were enrolled in the present research study. The overall median age of the female adolescents in the study was 17 years (IQR 16–17). The baseline length of menstruation was significantly greater (*p* = 0.009) for the aerobic exercise group (6.58 ± 1.03 days), followed by the stretching group (6.38 ± 1.13 days) and the aerobic plus stretching group (5.62 ± 1.30 days). Of the 78 research participants, half reported a regular menstrual bleeding pattern. A baseline summary and comparison of the characteristics of the research participants in each group is provided in Table 1.

### 3.2. Effectiveness of Exercise Programs on Length of Menstrual Cycle

A paired sample *t*-test was performed to establish the difference between pre-intervention and post-intervention length of menstrual cycle (days) for the 8-week three exercise programs (aerobic, stretching, and aerobic plus stretching). A small decrease in the length of the menstrual cycle was noted only for the aerobic exercise group (Mean Difference with 95% CI: 0.42 [0.06–0.79], *p* = 0.025) (Table 2).

### 3.3. Effectiveness of Exercise Programs on Menstrual Pain

To determine the difference between pre-intervention and post-intervention NRS scores for menstrual pain for all three exercise groups (aerobic, stretching, and aerobic plus stretching), a paired sample *t*-test was conducted (Table 2). There was a statistically significant reduction in menstrual pain score for the aerobic group (Mean Difference with 95% CI: 1.19 [0.20–2.18], *p* = 0.020), the stretching group (Mean Difference with 95% CI: 1.81 [0.95–2.67], *p* < 0.0001), and the aerobic plus stretching group (Mean Difference with 95% CI: 2.00 [1.25–2.75], *p* < 0.0001).

A between-groups analysis was conducted with ANCOVA to determine the significant difference in post-intervention mean menstrual pain score among the groups, with pre-intervention menstrual pain score and BMI as covariates. There was no significant effect of the different exercises on the level of menstrual pain after controlling for the effect of pre-intervention menstrual pain score and BMI (*p* = 0.090). Adjusted mean values of post-intervention menstrual pain score are provided in Table 3.

### 3.4. Effectiveness of Exercise Programs on Perceived Level of Stress

The difference between pre-intervention and post-intervention ASQ-S scores for perceived level of stress among the aerobic, stretching, and aerobic plus stretching exercise programs were analysed (Table 2). A statistically significant decline in stress scores was observed for the aerobic group (Mean Difference with 95% CI: 12.42 [4.78–20.06], *p* = 0.003) and the stretching group (Mean Difference with 95% CI: 13.73 [7.05–20.41], *p* < 0.0001).

ANCOVA was performed (Table 3), and there was no significant effect of the different exercise programs on perceived levels of stress after controlling for the effect of pre-intervention perceived stress score and BMI (*p* = 0.389).

### 3.5. Effectiveness of Exercise Programs on Adolescent Attitude towards Menstruation

Adolescent attitudes towards menstruation were assessed for differences in pre-intervention and post-intervention using AMAQ scores among the three exercise groups (Table 2). There was a statistically significant reduction in AMAQ scores post-intervention, indicating a negative impact on attitudes toward menstruation in all three exercises groups, with the highest mean difference observed in the aerobic plus stretching group (Mean Difference with 95% CI: 10.65 [3.70–17.60], *p* = 0.004), followed by the stretching group (Mean Difference with 95% CI: 7.89 [4.39–11.38], *p* < 0.0001), and the aerobic group (Mean Difference with 95% CI: 4.92 [0.74–9.11], *p* = 0.023).

In the ANCOVA analysis (Table 3), there was no significant effect of the different exercise programs on attitudes toward menstruation after controlling for the effect of pre-intervention attitude score and BMI (*p* = 0.825).

We executed a one-way ANOVA test to estimate the power based on the observed effect sizes among the groups. The analysis incorporated the means of menstrual characteristics for each group and a significance level (α) of 0.05. The results indicated that the study achieved a power of 93.8% for pain, 99.9% for stress, and 100.0% for attitude, suggesting that the sample size was adequate to detect significant differences between the groups.

## 4. Discussion

In this research study, we enrolled 78 participants and aimed to evaluate and compare the impact of aerobic, stretching, and aerobic plus stretching exercise programs on menstrual characteristics, including menstrual pain, stress, and attitudes toward menstruation in female adolescents in Saudi Arabia. Our findings suggested that participants in the aerobic exercise group experienced a significant reduction in the mean length of their menstrual cycle. Notably, the aerobic plus stretching group exhibited the most significant reduction in mean menstrual pain scores, while stress scores significantly decreased in the stretching group.

Pain is common during menstruation; however, its intensity can vary between individuals [25]. In the present study, we observed a significant reduction in NRS scores for all three exercises groups (aerobic exercise, stretching exercise, and a combination of aerobic plus stretching exercise), with the highest mean difference and pain relief noted in the aerobic plus stretching group. These findings align with a systematic review and network meta-analysis conducted by Tsai et al. (2024), which reported that various exercises effectively reduce the intensity and duration of pain in patients with primary dysmenorrhea [15]. Similar results were documented by Vaziri et al. (2015) for both aerobic and stretching exercises [18].

Regarding menstrual bleeding patterns, our findings indicated no significant difference across the three groups. However, a heavy menstrual bleeding pattern was prevalent in the stretching exercise group, while irregular bleeding was more common among participants in the aerobic exercise group. A study by Bruinvels et al. (2016) found that heavy menstrual bleeding is substantially prevalent among girls who exercise [26]. Conversely, another prospective study from Australia on a large cohort of women of reproductive age reported that physically active women had a decreased risk of heavy menstrual bleeding compared to those who reported no physical activity (OR 0.90, 95% CI 0.82–0.98) [27].

An increase in the frequency of uterine muscle contraction can significantly aggravate menstrual pain, as the uterine muscles receive nerve supply from the sympathetic nervous system. Stress has been reported to enhance sympathetic nervous system activity, intensifying menstrual pain by exacerbating uterine muscle contractions [12,13]. Another proposed mechanism for heightened pain is increased sensitivity. In a review study, Itani et al. (2022) highlighted that the underlying cause of severe dysmenorrhea could be high production of prostaglandin, leading to increased sensitivity to pain as a result of abnormalities in the pain pathway. The regular monthly menstrual cycle and inflammatory exposure of uterine muscles suggest that menstrual pain could also arise from heightened pain sensitivity [13].

Thus, painful menstruation involves both psychological and physical aspects. The ability of exercise to moderate stress by reducing the sympathetic nervous system activity may also contribute to the observed reduction in menstrual pain and related symptoms [28]. Furthermore, physical activities, such as exercising, lead to increased release of β-endorphins, endogenous opioid neuropeptides produced by the brain, which enhance the pain threshold and promote well-being [28].

In this study, we used the Adolescent Stress Questionnaire Short Version to assess stress among study participants. We observed significant effects from both aerobic and stretching exercises on alleviating stress in young adolescent girls. This reduction in stress may explain the decrease in menstrual pain observed in our study. Our findings are consistent with earlier systematic reviews of randomized controlled trials, which also confirmed that exercise can reduce stress levels [29].

The subject of menstruation remains stigmatized in numerous nations, with discussions surrounding menstruation often regarded as disgraceful. It is important to note that attitudes toward menstruation among young adolescent girls are heavily influenced by socioeconomic, cultural, and religious contexts. Moreover, knowledge also plays a critical role in shaping attitudes toward menstruation [30]. Poor awareness and misinformation regarding menstruation are associated with adverse attitudes and misconceptions about this natural physiological phenomenon, negatively impacting health and well-being [31].

In our study, we employed the Adolescent Menstrual Attitude Questionnaire to assess the attitudes of young adolescent girls toward menstruation. Our findings indicated a tendency toward negative attitudes across all three exercise groups, with the highest mean difference observed in the aerobic plus stretching exercise group. This finding may be attributed to female adolescents’ preference for remaining inactive during menstrual days.

In addressing this issue, proactive education of female adolescents about the importance and health benefits of maintaining an active lifestyle during menstruation could be beneficial. Emphasizing how physical activity during one menstrual cycle can significantly impact experiences in subsequent months could empower young girls with the knowledge and confidence needed to manage their menstrual health effectively.

### 4.1. Strengths and Limitations of the Study

This study compared the effectiveness of three different exercise programs (aerobic, stretching, and a combination), providing a comprehensive analysis of various physical activity types on menstrual characteristics. Assessments conducted during the first (pre-intervention) and third menstrual periods (post-intervention) allowed for a robust evaluation of the impact of different types of exercises over time. Focusing on healthy female adolescents aged 12 and 18 years provided valuable insights into a specific demographic that may benefit from exercise interventions.

However, the study involved a relatively small sample of 78 young adolescent girls from Riyadh province, Saudi Arabia, which may limit the generalizability of the findings to broader populations of reproductive-age girls in different age groups or residing in other provinces of Saudi Arabia. Larger sample sizes are typically preferred to enhance the reliability and applicability of study results across diverse populations. Additionally, the lack of a priori sample size determination may further impact the generalizability and statistical power of our results.

The recruitment of participants on a voluntary basis is another limitation of the current study, as this may introduce self-selection bias. Individuals who choose to participate may differ significantly from those who opt not to, potentially skewing the results. Furthermore, since participants were exclusively from Riyadh Province, the findings may not fully represent the diversity of cultural and socioeconomic backgrounds within Saudi Arabia. Therefore, caution is warranted when extrapolating these findings to girls from different regions or cultural settings within the country.

Moreover, variability in menstrual cycle length among the groups could affect the study’s outcomes and interpretations, highlighting a potential limitation. The study also did not assess dietary factors, despite evidence indicating that diet can influence menstrual pain, flow, and cycle length. Variations in dietary habits among participants could potentially confound the results related to menstrual characteristics and exercise interventions [32,33].

The findings were primarily based on self-reported data obtained through questionnaires (NRS, ASQ-S, AMAQ). While questionnaires are valuable tools for gathering subjective information, they are susceptible to recall bias and may not provide comprehensive insights into participants’ actual physical conditions or menstrual hygiene practices. Future studies could benefit from incorporating physical examinations or objective measures to validate questionnaire responses.

Finally, another limitation was the use of the English version of the ASQ-S. Although participants were proficient in English, the questionnaire had not been culturally adapted or validated for our population.

### 4.2. Recommendations

Future research should incorporate larger sample sizes and a more diverse population of female participants of reproductive age to enhance the generalizability and applicability of the findings. Additionally, future studies should emphasize the psychological aspects related to menstrual health. This focus will provide deeper insights into the influence of exercise on menstrual characteristics.

It is also essential to examine the impact of dietary habits and practices on menstrual pain, flow, and cycle length, as these factors can significantly influence menstrual experiences. Furthermore, investigating and comparing the effects of yoga and various levels of strength training on menstrual characteristics within our population would be beneficial, as both have been shown to alleviate premenstrual symptoms and dysmenorrhea, respectively [14,34,35].

## 5. Conclusions

In summary, this study observed a positive impact of both aerobic and stretching exercises in reducing menstrual pain, with aerobic exercise also contributing to a decrease in the length of menstrual days. Participants engaged in aerobic and stretching exercises demonstrated a reduction in stress levels. However, all three exercise groups exhibited a tendency towards a negative attitude towards menstruation, with the most significant mean difference noted in the group participating in both aerobic and stretching exercises.

Readers are encouraged to interpret these findings cautiously due to several important limitations. These include a small sample size, the absence of a priori sample size calculation, convenience sampling limited to the Riyadh region, variability in menstrual cycle length among groups, lack of dietary factor assessment, potential recall bias, and the use of the English version of the Adolescent Stress Questionnaire Short Version (ASQ-S), which was not culturally adapted for the study population.

## Figures and Tables

**Table 1:** Baseline characteristics of study participants (*n* = 78).

Variables	Total (*n* = 78)	Aerobic (*n* = 26)	Stretching (*n* = 26)	Aerobic Plus Stretching (*n* = 26)	*p* Value
Age (Years)	17 (16–17)	17 (16–17)	17 (15–18)	16 (15–17)	0.021 ^†^
BMI (Kg/m^2^)	22.88 ± 5.21	23.25 ± 5.04	23.65 ± 6.23	21.75 ± 4.17	0.060
Length of Menstruation (Days)	6.19 ± 1.22	6.58 ± 1.03	6.38 ± 1.13	5.62 ± 1.30	0.009
Menstrual Bleeding Pattern					
Regular	39 (50.0%)	12 (46.2%)	14 (53.8%)	13 (50.0%)	0.625 ^‡^
Irregular	28 (35.9%)	12 (46.2%)	7 (26.9%)	9 (34.6%)	
Heavy	11 (14.1%)	2 (7.7%)	5 (19.2%)	4 (15.4%)	
Physical Activity Level					
Light	36 (46.2%)	14 (53.8%)	13 (50.0%)	9 (34.6%)	0.546 ^‡^
Moderate	37 (47.4%)	10 (38.5%)	11 (42.3%)	16 (61.8%)	
Vigorous	5 (6.4%)	2 (7.7%)	2 (7.7%)	1 (3.8%)	
Numerical Rating Scale	5.69 ± 2.29	5.96 ± 2.38	5.92 ± 2.04	5.19 ± 2.45	0.400
Adolescent Stress Questionnaire Short Version	78.50 (61.75–90.00)	79.00 (63.50–92.25)	79.50 (68.25–85.00)	76.00 (52.25–90.25)	0.297 ^†^
Adolescent Menstrual Attitude Questionnaire	171.77 ± 11.04	168.96 ± 12.62	170.88 ± 7.31	175.46 ± 11.83	0.092

Note: ^†^ Kruskal–Wallis H Test, ^‡^ exact chi-square test, means ± standard deviations, medians and interquartile ranges, and frequencies and percentages were reported. Games–Howell Post Hoc Analysis (Age): Significant differences in age were observed only between aerobic and aerobic plus stretching groups (*p* = 0.037). Tukey Post Hoc Analysis (Adolescent Stress Questionnaire Short Version): No differences in Adolescent Stress Questionnaire Short Version scores were observed between any of the groups (*p* > 0.05).

**Table 2:** Paired comparison of exercise groups regarding pain, stress, and attitude among study participants (*n* = 78).

Variables	Aerobic (*n* = 26)			Stretching (*n* = 26)			Aerobic Plus Stretching (*n* = 26)		
	Mean ± SD	Mean Difference (95% CI)	*p* Value	Mean ± SD	Mean Difference (95% CI)	*p* Value	Mean ± SD	Mean Difference (95% CI)	*p* Value
Length of Menstrual Cycle (Days) *									
Pre-Intervention	6.58 ± 1.03	0.42 (0.06–0.79)	0.025	6.38 ± 1.13	-	-	5.62 ± 1.30	0.27 (−0.10–0.64)	0.148
Post-Intervention	6.15 ± 1.05			6.38 ± 1.13			5.35 ± 1.13		
Numerical Rating Scale									
Pre-Intervention	5.96 ± 2.38	1.19 (0.20–2.18)	0.020	5.92 ± 2.04	1.81 (0.95–2.67)	<0.0001	5.19 ± 2.45	2.00 (1.25–2.75)	<0.0001
Post-Intervention	4.77 ± 2.25			4.12 ± 1.66			3.19 ± 2.35		
Adolescent Stress Questionnaire Short Version									
Pre-Intervention	77.88 ± 15.50	12.42 (4.78–20.06)	0.003	76.15 ± 14.80	13.73 (7.05–20.41)	<0.0001	72.04 ± 22.08	5.62 (−1.48–12.71)	0.116
Post-Intervention	65.46 ± 18.26			62.42 ± 17.30			66.42 ± 23.12		
Adolescent Menstrual Attitude Questionnaire									
Pre-Intervention	168.96 ± 12.62	4.92 (0.74–9.11)	0.023	170.88 ± 7.31	7.89 (4.39–11.38)	<0.0001	175.46 ± 11.83	10.65 (3.70–17.60)	0.004
Post-Intervention	164.04 ± 12.37			163.00 ± 9.84			164.81 ± 12.33		

Abbreviations: SD, Standard Deviation; CI, Confidence Interval. * Paired comparison for Length of ‘Menstrual Cycle (Days)’ was not calculated for the ‘Stretching’ group because the standard error of difference was 0.

**Table 3:** Comparison of exercise groups for adjusted mean post-intervention scores (95% CI) for pain, stress, and attitude (*n* = 78).

Variables	Aerobic (*n* = 26)	Stretching (*n* = 26)	Aerobic Plus Stretching (*n* = 26)	*p* Value
Numerical Rating Scale *	4.62 (3.91–5.33)	3.97 (3.26–4.67)	3.49 (2.78–4.21)	0.090
Adolescent Stress Questionnaire Short Version ^†^	63.94 (57.44–70.45)	62.02 (55.51–68.52)	68.35 (61.77–74.93)	0.389
Adolescent Menstrual Attitude Questionnaire ^‡^	164.96 (160.67–169.24)	163.10 (158.85–167.36)	163.79 (159.42–168.16)	0.825

Abbreviation: CI, Confidence Interval. * Mean values were adjusted for pre-intervention Numerical Rating Scale score and BMI. **^†^** Mean values were adjusted for pre-intervention Adolescent Stress Questionnaire Short Version score and BMI. **^‡^** Mean values were adjusted for pre-intervention Adolescent Menstrual Attitude Questionnaire score and BMI.

## Data Availability

Data will be available upon request.
